# Enrichment of putatively damaging rare variants in the DYX2 locus and the reading-related genes *CCDC136* and *FLNC*

**DOI:** 10.1007/s00439-017-1838-z

**Published:** 2017-09-02

**Authors:** Andrew K. Adams, Shelley D. Smith, Dongnhu T. Truong, Erik G. Willcutt, Richard K. Olson, John C. DeFries, Bruce F. Pennington, Jeffrey R. Gruen

**Affiliations:** 1 Department of Genetics, Yale University, New Haven, CT USA; 2 Munroe Meyer Institute, University of Nebraska Medical Center, Omaha, NE USA; 3 Department of Pediatrics, Yale University, New Haven, CT USA; 4 Institute for Behavioral Genetics, University of Colorado, Boulder, CO USA; 5 Department of Psychology and Neuroscience, University of Colorado, Boulder, CO USA; 6 Department of Psychology, University of Denver, Denver, CO USA; 7 Department of Pediatrics and the Investigative Medicine Program, Yale University, New Haven, CT USA

## Abstract

Eleven loci with prior evidence for association with reading and language phenotypes were sequenced in 96 unrelated subjects with significant impairment in reading performance drawn from the Colorado Learning Disability Research Center collection. Out of 148 total individual missense variants identified, the chromosome 7 genes *CCDC136* and *FLNC* contained 19. In addition, a region corresponding to the well-known DYX2 locus for RD contained 74 missense variants. Both allele sets were filtered for a minor allele frequency ≤0.01 and high Polyphen-2 scores. To determine if observations of these alleles are occurring more frequently in our cases than expected by chance in aggregate, counts from our sample were compared to the number of observations in the European subset of the 1000 Genomes Project using Fisher’s exact test. Significant *P* values were achieved for both *CCDC136/FLNC* (*P* = 0.0098) and the DYX2 locus (*P* = 0.012). Taken together, this evidence further supports the influence of these regions on reading performance. These results also support the influence of rare variants in reading disability.

## Introduction

Reading disability (RD; dyslexia) is characterized by difficulty in reading despite normal intelligence and adequate access to educational opportunities. With a prevalence ranging from 5 to 17%, RD is the most common learning disability (Shaywitz and Shaywitz 2005). It persists throughout life, and has significant detrimental effects on educational achievement and long-term socioeconomic status (Schatschneider and Torgesen 2004). Twin and family studies show a large genetic component with estimates of heritability ranging from 0.34 to 0.76 (DeFries et al. 1987; Hawke et al. 2006). Despite this high heritability, studies have identified a limited number of associated genetic variants of small effect size.

**Fig. 1:**
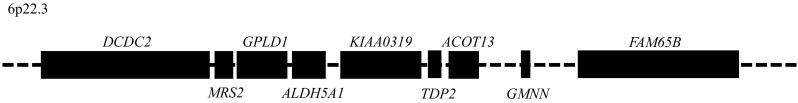
Depiction of the *DCDC2/KIAA0319* region sequenced. Gene sizes are proportional per sizes listed in human genome build GRCh37/hg19. Intergenic distances are approximate, total region length approximately 1.04 Mbp

The most frequently replicated associations with RD have been with two genes, *DCDC2* and *KIAA0319*, encoded in the DYX2 locus on 6p22 (Fig. 1) (Cardon et al. 1994; Carrion-Castillo et al. 2013; Cope et al. 2005; Gayán et al. 1999; Grigorenko et al. 1997; Harold et al. 2006; Meng et al. 2005; Schumacher et al. 2006; Turic et al. 2003). In addition to RD, *DCDC2* has been associated with specific language impairment and different reading and language phenotypes in diverse populations and languages (Poelmans et al. 2011). *KIAA0319* has been associated with both RD and general language performance phenotypes (Scerri et al. 2011). *DYX1C1*, encoded in the DYX1 locus on 15q, was first associated with RD by chromosomal breakpoint mapping in one family, with support from an association study on 23 additional families, 33 mixed status couples, and 100 population controls (Dahdouh et al. 2009; Taipale et al. 2003). Evidence for replication of other genes is weaker. *PCDH9*, encoded on 13q21.32, falls within a linkage peak first associated with RD in a multipoint linkage analysis (Bartlett et al. 2002). *SEMA6D*, encoded on 15q21.1, was identified by chromosomal breakpoint mapping in one subject with RD (Ercan-Sencicek et al. 2012). *COMT*, encoded on 22q11.21, has been associated with several reading-related phenotypes (Grigorenko et al. 2007; Landi et al. 2013). *CCDC136*, *FLNC* (encoded on 7q32.1), and *RBFOX2* (encoded on 22q12.3) were associated with reading performance in a genome-wide association study (GWAS) of 1862 subjects selected from three family-based cohorts (Gialluisi et al. 2014).

RD frequently co-occurs with specific language impairment (SLI), defined as delayed onset of language acquisition, often including impairment in receptive vocabulary. 43% of children with SLI develop RD when they are later exposed to a reading curriculum in school (Snowling et al. 2000). The prevalence of SLI in kindergarten age children from the United States is approximately 7% (Tomblin et al. 1997). Like RD, the heritability for SLI is high, ranging from 0.36 to 0.97 in twin studies (Bishop and Hayiou-Thomas 2008). While there have been several linkage and association studies of SLI, evidence for two genes, *FOXP2* and *CNTNAP2*, are the most compelling. Through a series of chromosomal breakpoint mapping studies performed on a large, multigenerational family (called “KE”), *FOXP2* was initially found to be associated with verbal dyspraxia, a type of SLI (Fisher et al. 1998). *CNTNAP2* has been associated with nonsense word repetition in 184 families with a history of SLI. Nonsense word repetition is a quantitative assessment of phonological processing, which is frequently impaired in SLI (Bishop et al. 1996). Both *FOXP2* and *CNTNAP2* were associated with nonsense word repetition in a sample of 188 dyslexic families (Peter et al. 2011). Interestingly, FOXP2 is a transcription factor expressed in brain that binds and regulates expression of *CNTNAP2* (Vernes et al. 2008). The regulatory interaction between the FOXP2 protein and *CNTNAP2* supports the clinical findings that both genes are involved in similar reading and language phenotypes.

Attention deficit hyperactivity disorder (ADHD) also commonly co-occurs with RD. 80% of individuals with ADHD meet diagnostic criteria for at least one additional learning disorder; 40% of males with RD also have ADHD (Germano et al. 2010; Willcutt and Pennington 2000). While several genes have previously been associated with ADHD, *DBH* and *DRD2* have also been associated with reading and language (Smith et al. 2003). *DRD2* was associated with ADHD in a structural equation modeling study using 236 subjects, while later studies showed association with verbal language (Eicher et al. 2013; Rowe et al. 1999).

Previous association studies have focused on common variation, which accounts for only a small portion of the previously described heritability for RD. As costs for sequencing continue to fall, rare variants have recently become of interest, because they generally have larger effect sizes than non-coding SNPs, and they explain a larger portion of the observed heritability in complex traits. Projects such as the 1000 Genomes Project (The Genomes Project 2015), Exome Aggregation Consortium (ExAC) (Lek et al. 2016), and more recently the genome Aggregation Database (gnomAD; Lek et al. 2016) have catalogued a large portion of the genetic variation present in human populations at low frequency, protein altering, locations which is critical for determining whether they are enriched in any sample selected for a particular trait.

Resequencing studies have tended to show an enrichment of rare variants relative to controls in genes previously identified by GWAS. For example, in a 2010 study of hypertriglyceridemia (HTG), Johansen et al. demonstrated an enrichment of rare, putatively damaging mutations, with a 3-to-6-fold enrichment of missense and nonsense mutations in *GCKR*, *APOB*, *LPL*, and *APOA5* in 438 cases compared to 327 controls. These genes were initially identified by a GWAS of 463-affected subjects (and 1197 controls) predicated on a diagnosis of Fredrickson hyperlipoproteinemia as a categorical trait (Johansen et al. 2010).

To date, there have been two rare variant studies of RD. One study used imputed rare variants to study mismatch negativity in 200 children with RD, and found an association with four variants in DYX2 located midway between *DCDC2* and *KIAA0319* (Czamara et al. 2011). A second study of 376 subjects focused on copy number variants, but demonstrated no significant difference in the burden of CNVs between cases with RD and non-RD controls (Girirajan et al. 2011).

In this study, we seek to utilize high-throughput deep sequencing in 96 unrelated individuals with RD to investigate whether there is an enrichment in rare, putatively damaging variants in genes previously associated with reading or language performance by linkage and association analyses.

## Methods

### Subjects

Subjects were selected from the Colorado Learning Disability Research Center (CLDRC) nuclear family collections assessed for reading performance. The CLDRC maintains a collection of DNA samples from over 800 RD families phenotyped with a wide array of reading and language performance assessments (DeFries et al. 1997). For sequencing, 96 affected, unrelated subjects of European ancestry were selected based on a CLDRC-derived discriminant score indicating impairment in reading ability. Cases were defined as having a discriminant score of less than −0.60. Discriminant score coefficients were generated from performance on the spelling, reading comprehension, and the reading recognition subtests of the peabody individual achievement test. Discriminant function coefficients were then used to calculate a quantitative score used to maximally differentiate RD subjects from the general population (Wadsworth et al. 1989). Scores ranged from −0.60 to −4.65, with a mean of −2.04. Sixty-three subjects were male and 33 subjects were female, with ages ranging from 8.14 to 17.53 years at testing (mean 11.63). Fifteen included subjects were comorbid for ADHD.

All protocols were reviewed and approved by relevant Institutional Review Boards at the University of Colorado, University of Nebraska Medical Center, and Yale University. Subjects and families provided informed consent for use of both phenotype and genetic information at time of sample collection. In the absence of sequencing from non-RD controls, variant counts were compared to the European subset of the 1000 Genomes Project (1KGE) through the publicly available browser hosted by Ensembl (http://www.ensembl.org). 1KGE participants were apparently normal individuals, but were not specifically screened for reading or language phenotypes (The Genomes Project 2015).

### Target selection and sequencing

Eleven genomic regions were targeted for a total of 6.4 Mb sequenced. Regions for sequencing were selected based on published genetic association and/or preliminary association with selected quantitative measures of reading performance on the full CLDRC sample (Table 1). Briefly, a targeted association analysis was performed in 1428 individuals from 337 families using 332 SNPs contained within 26 candidate genes plus 1 kbp on each side to include UTRs. Phenotypes for analysis included individual and composite measures of word reading, rapid automatized naming, orthographic coding, and comprehension. Seven genes contained one or more SNPs that survived correction for multiple testing with the False Discovery Rate method, with corrected *P* <0.05 for at least one phenotype (manuscript in preparation). Results from this analysis (including both uncorrected and corrected *P* values) were combined with literature support to obtain the final list of genes for sequencing. *CNTNAP2*, *COMT*, *DBH*, *DCDC2*-*KIAA0319*, *DRD2*, *DYX1C1*, and *FOXP2* had nominally significant results (uncorrected *P* < 0.05) for multiple SNPs in the CLDRC. Association with multiple SNPs from the *CCDC136/FLNC* locus and *RBFOX2* was described in a published GWAS that included 729 CLDRC subjects, including 51 of our affected subjects (Gialluisi et al. 2014). *PCDH9* and *SEMA6D* were included from extant publications (Bartlett et al. 2002; Ercan-Sencicek et al. 2012). Support was limited for other RD candidates including *C2ORF3* and *ROBO1* in the preliminary association analysis and was excluded for this reason. *CYP19A1* was excluded, because the previous analysis of multiple SNPs in this gene in the CLDRC cohort did not show association (Anthoni et al. 2012).

**Table 1:** Loci sequenced for this study

Locus	Chr. Loc.	Sequenced coordinates	Size (Mb)	Rationale for selection
*DCDC2/KIAA0319*	6p22.3	24,124,528–25,166,900	1.04	Reading candidate^a^ (Meng et al. 2005)
*FOXP2*	7q31.1	113,708,455–114,351,800	0.643	Reading candidate^a^ (Peter et al. 2011)
*CCDC136/FLNC*	7q32.1	128,417,100–128,501,990	0.085	Reading candidate^a^ (Gialluisi et al. 2014)
*CNTNAP2*	7q35-q36.1	145,792,593–148,183,542	2.4	Reading and language candidate^a,b^ (Vernes et al. 2008)
*DBH*	9q34.2	136,498,622–136,524,722	0.026	Preliminary association with reading (data not shown)
*DRD2*	11q23.2	113,279,322–113,350,600	0.071	Language candidate^b^ (Eicher et al. 2013)
*PCDH9*	13q21.32	66,843,132–67,813,032	0.97	Language candidate^b^ (Bartlett et al. 2002)
*SEMA6D*	15q21.1	47,458,034–48,078,585	0.628	Language candidate^b^ (Ercan-Sencicek et al. 2012)
*DYX1C1*	15q21.3	55,647,446–55,819,300	0.303	RD candidate^c^ (Taipale et al. 2003)
*COMT*	22q11.21	36,129,602–36,432,802	0.309	Reading and language candidate^a,b^ (Grigorenko et al. 2007)
*RBFOX2*	22q12.3	19,922,500–19,961,200	0.172	Reading candidate^a^ (Gialluisi et al. 2014)

Each locus is named for the major associated gene(s) present. Columns are chromosomal location, sequenced coordinates in NCBIv37/hg19, approximate size of the locus in Mb, and rationale for selection. Genes selected based on literature support include corresponding citation

^a^ Association with reading component phenotype or preliminary association evidence

^b^ Association with a language phenotype or SLI

^c^ Association with RD as a categorical trait

DNA sequencing was performed at the Yale Center for Genome Analysis (YCGA). Regions for sequencing were isolated and amplified with the Roche NimbleGen SeqCap EZ Target capture protocol. NimbleGen SeqCap is a system that allows for the isolation and amplification of a library corresponding to targeted sequence regions (Roche Sequencing, Pleasanton, CA). Libraries were sequenced on an Illumina Hi-Seq 2500 generating 75 bp, paired end reads (Illumina, San Diego, CA). Base calls and corresponding quality scores were generated by the YCGA.

### Variant identification and analysis

Unaligned reads with corresponding quality scores were retrieved from the YCGA in FASTQ format. Downstream analysis of sequence data was performed on the Yale high-performance computing cluster in a Linux environment using an in-house sequence analysis pipeline modified for the analysis of targeted regions (ycga.yale.edu). Reads were first aligned to human reference genome build NCBI37 (hg19) using the Burrows–Wheeler Aligner memory efficient algorithm (bwa-mem) (Li 2013; Li et al. 2009). Aligned BAM files were then processed according to the Broad Institute’s best practices for sequence data analysis. The Genome Analysis Toolkit (GATK) HaplotypeCaller identified variants from the reference genome (DePristo et al. 2011; McKenna et al. 2010). Because of the limited information provided by the size of the targeted regions, hard filtering instead of variant quality score recalibration was applied to variants (McKenna et al. 2010). A VCF file containing variants was downloaded from the Yale computing cluster and read with SNP and Variation Suite version 8 (Golden Helix, Inc., Bozeman, MT). Variants altering protein sequence (counts are reported in Table 2) were filtered for only those with minor allele frequencies (MAF) of less than 1%. Variant allele frequencies were determined using both the European subset of the 1KGE and the non-Finnish European subset of the gnomAD database (Tables 3, 4). Novel variants were also included in downstream analyses (Table 3). The remaining variants were assayed for putative damage to the corresponding proteins using SIFT (Kumar et al. 2009). Variants were further filtered based on Polyphen-2 scores >0.7, indicating a high likelihood of significant damage to protein function (Adzhubei et al. 2010). The higher the Polyphen-2 score, the more likely the variant is damaging to the protein, with scores ranging from 0 to 1. Variant counts in RD case subjects were compared to counts of the same variants in the 1KGE using a two-tailed Fisher’s exact test implemented in R (Team R Core 2016).

**Table 2:** Observed missense variant counts in each sequenced locus

Locus	# of Genes	# of Variants
*DCDC2/KIAA0319*	9	74
*FOXP2*	1	3
*CCDC136/FLNC*	2	19
*CNTNAP2*	1	2
*DBH*	1	10
*DRD2*	1	7
*PCDH9*	1	7
*SEMA6D*	1	6
*DYX1C1*	2	10
*COMT*	2	8
*RBFOX2*	4	2
Total	25	148

Loci are named for the major associated gene(s) present. The number of genes in each individual locus is also reported

**Table 3:** Variants in *CCDC136* and *FLNC* with MAF <0.01 and PolyPhen-2 scores greater than 0.75

Variant (11 total)	Chr	Position	Gene	Rs ID	AA change	SIFT	PolyPhen	CDS Obs	1KGE Obs	gnomAD
7:128441492-SNV	7	128441492	*CCDC136*	rs185493260	Arg/His	0	1	3	5	2.43 × 10^−3^
7:128434498-SNV	7	128434498	*CCDC136*	rs143537881	Gly/Asp	0.01	0.923	2	6	1.03 × 10^−2^
7:128449696-SNV	7	128449696	*CCDC136*	rs142055253	Lys/Glu	0	0.792	1	3	2.38 × 10^−3^
7:128446877-Frameshift	7	128446877	*CCDC136*	Novel	N/A	N/A	N/A	1	0	N/A
7:128483880-SNV	7	128483880	*FLNC*	rs768103657	Gly/Arg	0	1	1	0	7.41 × 10^−5^
7:128480184-SNV	7	128480184	*FLNC*	rs189525930	Gly/Arg	0	0.981	1	3	1.29 × 10^–^^3^
7:128485240-SNV	7	128485240	*FLNC*	rs146953558	Arg/Cys	0.02	0.953	1	7	9.52 × 10^−3^
7:128483367-SNV	7	128483367	*FLNC*	rs374983276	Arg/Cys	0	0.932	1	0	2.48 × 10^−4^
7:128491324-SNV	7	128491324	*FLNC*	rs181067717	Arg/Cys	0.01	0.901	2	5	8.17 × 10^−3^
7:128481578-SNV	7	128481578	*FLNC*	rs34972246	Asp/Ala	0	0.898	1	5	4.97 × 10^−3^
7:128494547-SNV	7	128494547	*FLNC*	rs202223616	Glu/Gln	0.11	0.81	1	1	1.15 × 10^−3^
							Variant	15	35	
							Reference	177	971	

Each variant is listed with chromosome, genomic location in hg19 coordinates, gene, rsID (if one has been assigned), SIFT score, PolyPhen-2 score, number of observations in our RD data set (CDS), number of observations in European subjects (*N* = 503) from the 1000 Genomes Project, and allele frequency in the gnomAD database. Amino acid changes are presented using the standard three-letter codes as reference/alternate. Variants without an rsID are novel to this study. Allele counts (variant and reference) are presented at the bottom of the table. Two-tailed Fisher’s exact test *P* value = 0.0098

**Table 4:** Variants in *DCDC2/KIAA0319* locus with MAF <0.01 and PolyPhen-2 scores greater than 0.75

Variant (15 total)	Chr	Position	Gene	Rs ID	AA change	SIFT	PolyPhen	CDS Obs	1KGE Obs	gnomAD
6:24520644-SNV	6	24520644	*ALDH5A1*	rs149482918	Ala/Thr	0.03	0.986	1	0	7.89 × 10^−6^
6:24520719-SNV	6	24520719	*ALDH5A1*	rs115784602	Val/Met	0.01	0.955	1	4	4.96 × 10^−3^
6:24178856-SNV	6	24178856	*DCDC2*	rs766600237	Pro/Leu	0.03	0.874	1	0	3.17 × 10^−5^
6:24174966-SNV	6	24174966	*DCDC2*	rs145154884	Val/Met	0	0.791	1	1	5.53 × 10^−5^
6:24848281-SNV	6	24848281	*FAM65B*	rs375981215	Thr/Lys	0	1	1	0	4.73 × 10^−5^
6:24495234-SNV	6	24495234	*GPLD1*	rs200793796	Cys/Gly	0.02	0.991	2	10	9.39 × 10^−3^
6:24472814-SNV	6	24472814	*GPLD1*	rs62400471	Arg/Cys	0.02	0.912	1	1	6.39 × 10^−4^
6:24476431-SNV	6	24476431	*GPLD1*	rs61754637	Asn/Ser	0.04	0.771	1	3	2.10 × 10^−3^
6:24559241-SNV	6	24559241	*KIAA0319*	rs773691612	Gly/Arg	0.24	0.956	1	0	3.24 × 10^−5^
6:24596526-SNV	6	24596526	*KIAA0319*	rs148566925	Pro/Thr	0.06	0.869	1	4	4.87 × 10^−3^
6:24566953-SNV	6	24566953	*KIAA0319*	rs113411083	Arg/Trp	0.08	0.835	1	6	4.68 × 10^−3^
6:24596675-SNV	6	24596675	*KIAA0319*	rs201302072	Arg/Leu	0.15	0.778	1	0	1.58 × 10^−4^
6:24412567-SNV	6	24412567	*MRS2*	rs200245617	Val/Ile	0.12	0.973	1	0	4.39 × 10^−4^
6:24667009-SNV	6	24667009	*TDP2*	rs146127613	Leu/Val	0.01	0.923	1	4	3.00 × 10^−3^
6:24667074-SNV	6	24667074	*TDP2*	rs61760186	Cys/Tyr	0.35	0.781	1	5	4.47 × 10^−3^
							Variant	16	38	
							Reference	176	968	

Each variant is listed with chromosome, genomic location in hg19 coordinates, gene, rsID (if one has been assigned), SIFT score, PolyPhen-2 score, number of observations in our RD data set (CDS), number of observations in European subjects (*N* = 503) from the 1000 Genomes Project, and allele frequency in the gnomAD database. Amino acid changes are presented using standard three-letter codes as reference/alternate. Variants and reference alleles are presented as counts out of number of observed alleles. Two-tailed Fisher’s exact test *P* value = 0.0118

### Linkage disequilibrium

**Fig. 2:**
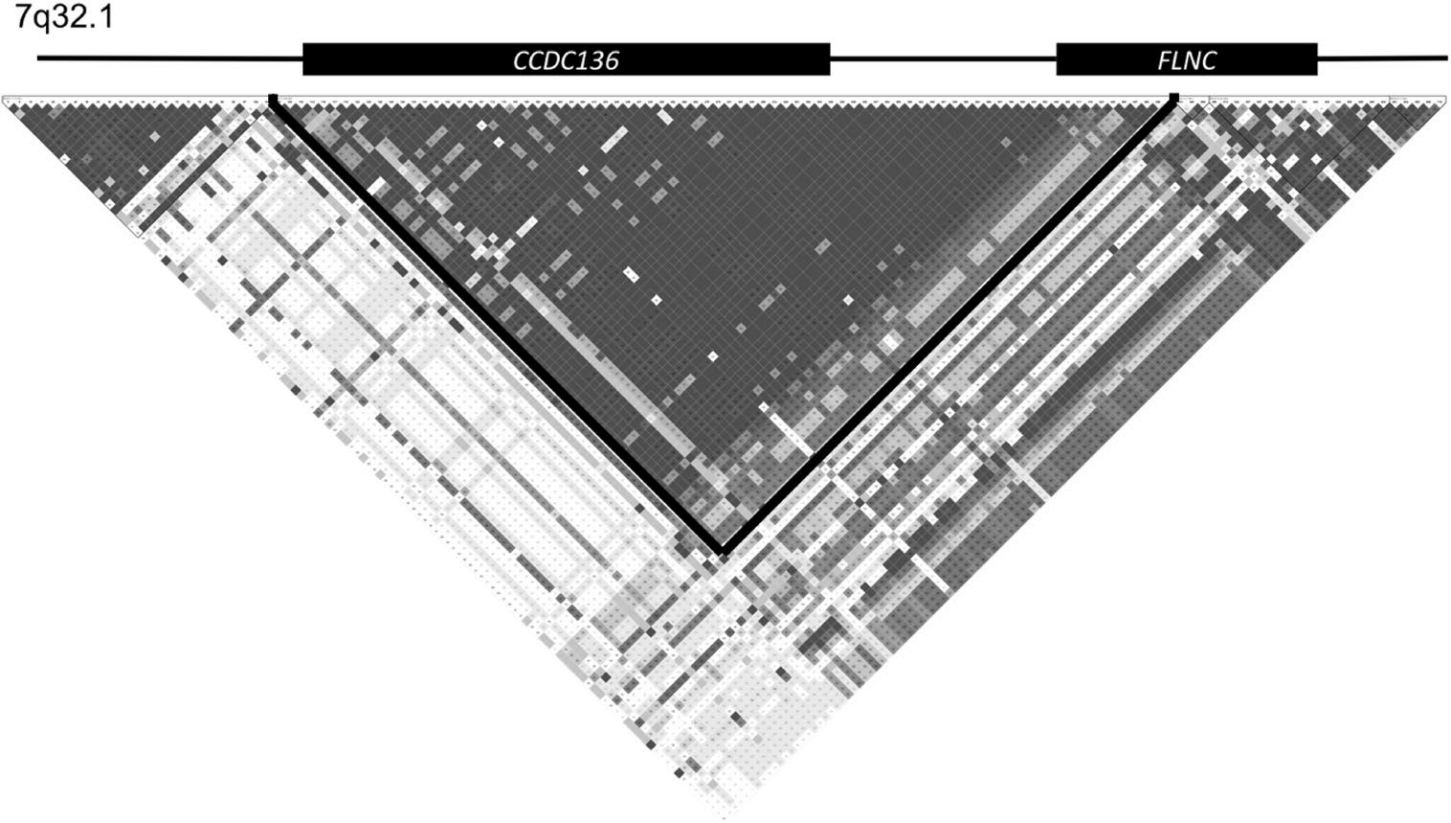
Heatmap of LD across *CCDC136/FLNC*. Coloring is based on D’ values with *darker gray* indicating high LD between marker pairs. Genes are aligned to markers on the heatmap, but are not accurately scaled. The major LD block joining *CCDC136* and *FLNC* is outlined in *black*

Data for the *CCDC136/FLNC* region from the 503 European subjects from the 1KGE were downloaded from the 1000 Genomes Project data browser hosted at Ensembl. (http://www.ensembl.org). Data were loaded into Haploview and D’ was calculated for all pairwise comparisons of markers (Barrett et al. 2005). Heatmaps were generated (Fig. 2), with LD blocks determined using the solid spine of LD method, filtering out variants with a minor allele frequency of less than 0.05.

## Results

### Variants

In total, 148 missense variants were identified in 96 RD cases. Within the 11 sequenced regions, two regions contained a disproportionate number of single nucleotide variants (SNVs) (Table 2). *CCDC136/FLNC* encoded on chromosome 7 contained 19 missense variants. The *DCDC2*–*KIAA0319* region (roughly contiguous with the DYX2 locus) encoded on chromosome 6p22 contained 74 missense variants.

To restrict the analysis to rare SNVs, only those with MAF ≤0.01 were considered in downstream analyses. Ten of the eleven variants with MAF ≤0.01 in *CCDC136/FLNC* (Table 3) were previously observed missense mutations causing putatively damaging changes to the relevant protein with Polyphen-2 scores ranging from 0.792 to 1. A Polyphen-2 score greater than 0.70 was generally considered damaging to proteins. Four variants were observed in *CCDC136*, including a one-base frameshift variant (7:128446877-Frameshift) not found in the 1KGE. The remaining seven variants were found in *FLNC* with Polyphen-2 scores ranging from 0.81 to 1. Within DYX2, there were 16 observations of 15 variants with an MAF ≤0.01 and Polyphen-2 scores ranging from 0.771 to 1 (Table 4). Six of these 15 variants were not observed in the 1KGE, but were observed in the gnomAD database and are annotated in dbSNP (Table 4).

### Enrichment analysis

Variant counts found in Tables 3 and 4 were used to test for enrichment of rare, damaging variants in cases relative to 1KGE. Within the *CCDC136/FLNC* region, 15 out of 192 chromosomes (7.8%) in RD cases contained one of the rare putatively damaging variants, compared to 35 out of 1006 chromosomes (3.5%) in the 1KGE, *P* = 0.0098 by two-tailed Fisher’s exact test (Table 3). Within the DYX2 region, 16 out of 192 chromosomes (8.3%) in RD cases contained one of the rare putatively damaging variants, compared to 38 out of 1006 chromosomes (3.8%) in the 1KGE, *P* = 0.012 by two-tailed Fisher’s exact test (Table 4).

Number of variants per subject ranged from 0 to 7, with no consistent correlation between discriminant score and number of variants. Eighteen subjects had no variants, 17 had one variant, 25 subjects had two variants, 14 subjects had three variants, 14 subjects had four variants, five subjects had five variants, two subjects had six variants, and one subject had seven variants. The worst performing subject had 0 variants that met all filtering criteria, and the subject with the most variants had a discriminant score of −2.41.

## Discussion

In this study, 11 loci were sequenced in 96 unrelated RD subjects with significant impairment in reading performance. The loci were chosen, because they were previously associated with RD in the literature and/or in a preliminary association analysis (data not shown). In total, 148 missense variants were observed. Both the *CCDC136/FLNC* region and DYX2 locus showed a significant enrichment for rare (MAF ≤0.01), putatively damaging SNVs.

### *CCDC136/FLNC*

A subset of 51 CLDRC RD subjects from this study was used in the published GWAS (total *N* = 1862) predicated on the first principal component from several quantitative reading and language exams (Gialluisi et al. 2014). This GWAS was the first to demonstrate association between *CCDC136/FLNC* and reading or language performance, with a weak, but replicated association between rs59197085 and both genes.

*CCDC136* and *FLNC* are tandemly encoded in a genomic segment of just 48,000 bps that span four blocks of linkage disequilibrium (Fig. 2). The central block of linkage disequilibrium, however, spans the entirety of *CCDC136* and the 5′ half of *FLNC*, making it difficult to distinguish genetic association from one gene or the other. This justifies considering both genes as a single genetic locus in the previous GWAS as well as in the current study.

*CCDC136* (also known as *NAG6*) is a coiled-coil domain containing protein described as a potential tumor suppressor in gastric cancer (Jiang et al. 2000). There is evidence for brain expression in both the GTEx and Brainspan databases. In GTEx, human cortex has a median RPKM value (a normalized value indicating transcript abundance) of 14.250 based on an analysis of 114 human samples, while cerebellum has a median RPKM of 27.700 based on 125 human samples (Lonsdale et al. 2013). Brainspan demonstrates evidence for expression in humans, beginning at approximately 8 postconception weeks (pcw) and continuing throughout life (BrainSpan 2011). Data from the ExAC project suggest that *CCDC136* is fairly mutation tolerant with 314 observed missense variants compared to an expected 283.1, corresponding to a non-significant constraint metric of −0.90 (Lek et al. 2016).

*FLNC* (Filamin C) is a cytoskeletal component, responsible for crosslinking actin. Filamins are especially prominent in muscle, including cardiac muscle. Previous studies have implicated *FLNC* in a collection of myopathies and familial cardiomyopathies (Brodehl et al. 2016; Duff et al. 2011; Jiang et al. 2000; Shatunov et al. 2009; Thompson et al. 2000; Valdes-Mas et al. 2014; Vorgerd et al. 2005; Williams et al. 2005). Evidence for brain expression is limited, especially relative to *CCDC136*, with no expression observed in GTEx and only limited expression in Brainspan. Only a single sample in Brainspan demonstrates evidence for neuronal expression occurring at approximately 8–9 pcw, with expression tapering off to relatively low levels postnatally. ExAC missense variant tolerance information suggests that *FLNC* is not variation tolerant with an observed 865 missense variants compared to an expected 1191.1, for a corresponding constraint metric of 4.62.

Comparing the contrasting expression and functional evidence for both genes described above, it is unclear which gene may be playing the more important role in RD. Due to sample size and regional/timepoint specification, making conclusions solely based on Brainspan and/or GTEx is difficult. *FLNC* may be expressed at a non-assayed timepoint or brain location in development, and thus not observed in either database. The mutation tolerance data from ExAC suggest that variants in *FLNC* are actively being removed from the genome, though whether this is due to the known cardiac consequences of *FLNC* mutations or an unknown role in language processing is not clear. *CCDC136* shows evidence for brain expression but no significant evidence for mutation intolerance. Further study is required to disentangle the role of each gene on reading performance.

### *DCDC2/KIAA0319*

DYX2 (contiguous with the sequenced *DCDC2/KIAA0319* region), the second genomic locus associated with RD, was first identified in a 1994 QTL study by Cardon et al. that utilized 27 of the same RD twinships/families in the current study (Cardon et al. 1994). Within DYX2, two genes, *DCDC2* and *KIAA0319*, have demonstrated consistent association with reading performance (as a quantitative trait) or RD (as a categorical trait). Knockout or knockdown studies of specific DYX2 genes in rodent models have demonstrated defects in auditory discrimination and neural spike timing, suggesting possible pathophysiologic mechanisms (Centanni et al. 2016; Che et al. 2014). RNAi studies in embryonic rats have shown that disruption of *Dcdc2* leads to neuronal migration defects (Burbridge et al. 2008). *DCDC2* localizes to cilia, and has been implicated in both autosomal (dominant or recessive) human kidney disease and autosomal recessive deafness (Massinen et al. 2011; Schueler et al. 2015). Typically, RD presents with a complex inheritance pattern indicative of a polygenic disorder. Also of note is that our group and others have shown that READ1 (Regulatory Element Associated with Dyslexia 1), a transcription regulatory element encoded in the second intron of *DCDC2*, is strongly associated with reading performance (Powers et al. 2013). Mass spectroscopy studies (SILAC) show that the transcription factor ETV6 binds READ1 sequence, while chromatin conformation capture (3C) experiments demonstrate close physical proximity between READ1 and the nearby *KIAA0319* promoter (KIAHap) in cultured human cells (Powers et al. 2016). *KIAA0319* encodes a membrane protein with a highly glycosylated extracellular domain suspected to play a role in signaling and neuronal adhesion (Velayos-Baeza et al. 2010). Evidence for human brain expression is strong in both GTEx and Brainspan. *KIAA0319* is expressed across all developmental timepoints in Brainspan, and has median RPKM values of 5.898 in the cortex (*N* = 114) and 5.996 in the cerebellum (*N* = 125). *DCDC2* has limited evidence for expression in Brainspan. Additional genes within the DYX2 locus (falling between *DCDC2* and *KIAA0319*) have shown varying degrees of association, though whether this is a true association or a consequence of linkage disequilibrium in the region is unknown.

It is probable that unrecognized RD is present in 1KGE subjects, which would inflate the observed variant counts among controls and diminish the significance in RD cases. Assuming that the 1KGE are representative of the general population (reading performance is a normally distributed trait), we would expect some of the 1KGE to be as impaired as our average case subject. Using controls screened for reading performance would likely strengthen the observed enrichments, but probably not by very much, because as noted above, we selected for less frequent extremes of the RD phenotype as cases. However, this may limit the generalizability of our results. For this study, utilizing subjects from a single ethnic group minimizes confounding due to population-level differences in the frequency of rare variants.

There is a limited, but growing, body of evidence in support of rare variants playing a role in RD. In a 2011 study, Czamara et al. demonstrated an association between late mismatch negativity (a neural trait shown to be affected in RD cases) and four imputed rare variants (three with significant LD to one another) in the DYX2 region (Czamara et al. 2011). Further studies of rare forms of variation, including CNV studies, have been inconclusive, showing no significant difference between cases and controls in global CNV burden (Girirajan et al. 2011). Our results add to the evidence that rare variants are an important consideration in the genetic etiology of RD, and deserve further study in both family and population designs. Additional study may reveal that reading performance hinges on a complex mix of genetic variants ranging from rare pseudo-Mendelian variants having large effects (typically through disrupting proteins) to common non-coding variants affecting gene expression or cryptic non-coding RNAs.

Previous association studies have revealed few functional (protein altering) variants associated with complex traits. Most variants identified through GWAS occur outside of coding regions of genes and have no clear functional consequences. This has led to two main hypotheses over the meaning of GWAS results. The first is that GWAS-associated SNPs are found in undescribed or undefined regulatory regions, acting to subtly alter transcription or splicing of nearby associated genes. The second hypothesis, and the one investigated in this study, is that common variants typically used in GWAS are acting to tag a collection of rare variants in coding regions that are either not genotyped, or excluded from other analyses due to low minor allele frequencies in the general population. These rare variants change the protein-coding sequence and provide the “true” effect leading (at least in part) to the phenotype upon which the GWAS was predicated (Manolio et al. 2009). While no single variant can be associated with RD based on this study due to sample size limitations, there is evidence of a statistically significant increase in the frequency of specific observed mutations in RD cases.
